# Active Components of Traditional Chinese Medicinal Material for Multiple Myeloma: Current Evidence and Future Directions

**DOI:** 10.3389/fphar.2022.818179

**Published:** 2022-01-27

**Authors:** Chao-Chao Yu, Yi Li, Zhao-Jun Cheng, Xi Wang, Wei Mao, Ying-Wen Zhang

**Affiliations:** ^1^ Department of Integrated Chinese and Western Medicine, Zhongnan Hospital of Wuhan University, Wuhan University, Wuhan, China; ^2^ Department of Oncology, Zhongnan Hospital of Wuhan University, Wuhan University, Wuhan, China; ^3^ Department of Spinal Surgery, The First Affiliated Hospital of Guangzhou University of Chinese Medicine, Guangzhou, China; ^4^ Department of Oncology, Wuhan Hospital of Traditional Chinese Medicine, Wuhan, China; ^5^ Peking University Shenzhen Hospital Hua Wei Clinic, Shenzhen, China

**Keywords:** multiple myeloma, traditional Chinese medicinal materials, bioactive components, pharmacological effects, review

## Abstract

Multiple myeloma (MM) is a hematological malignancy characterized by clonal expansion of plasma cells in bone marrow, leading to the overproduction of monoclonal immunoglobulins. The clinical manifestations resulting from monoclonal proteins and malignant cells include signs of end-organ damage, such as hypercalcemia, renal failure, anemia, and bone lesions. Despite improvement in the survival of MM patients with use of myeloma-targeted and immunomodulatory therapies, MM remains an incurable disease. Moreover, patients with relapsed or refractory MM show poor survival outcomes. In recent years, there has been a growing interest in the use of traditional Chinese medicinal materials (TCMMs) for management of a wide spectrum of diseases. The bioactive ingredients derived from TCMMs hold great potential for the development of anticancer drugs. Here we summarize the evidence of the pharmacological effects of the active components in TCMMs on MM, including curcumin, resveratrol, baicalein, berberine, bufalin, cinobufagin, gambogic acid, ginsenoside, icariin, daidzin, formononetin, polysaccharides extracts from *Hedyotis difus*, and scutellarein. Available evidence indicates that the anti-MM effects of these bioactive ingredients are mediated via regulation of proliferation, apoptosis, autophagy, cell cycle, osteogenic differentiation, and drug resistance. In the future, the underlying mechanisms of the anti-MM effects of these components should be further investigated. Large-scale and well-designed clinical trials are also required to validate the efficacy of these bioactive constituents for MM.

## Introduction

**FIGURE 1:**
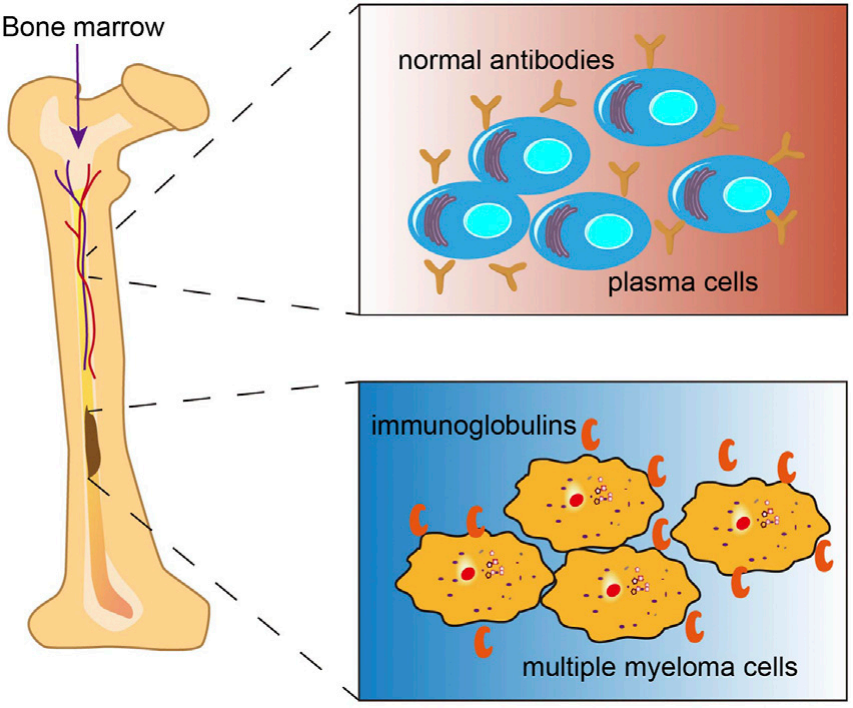
Multiple myeloma (MM). MM is a malignancy of plasma cells originating from the bone marrow, which produces excess monoclonal immunglobulins.

Multiple myeloma (MM) is the second most commonly diagnosed hematologic malignancy, accounting for nearly 10% of all hematological malignancies (Kumar et al., 2017). Environmental and occupational exposure (Georgakopoulou et al., 2021), genetic factors, and epigenetic alterations (Dimopoulos et al., 2014), race (Marinac et al., 2020) have been implicated in the causation of MM(Rajkumar, 2020). The disease is characterized by progressive monoclonal proliferation in the bone marrow, which leads to the overproduction of nonfunctional intact immunoglobulins (also known as M protein or monoclonal protein) (González et al., 2007; Joshua et al., 2019) or immunoglobulin chains in 15–20% of patients (Figure 1) (Drayson et al., 2001). Accumulation of these immunoglobulins and interaction of the aberrant monoclonal plasma cells with other cells in the bone marrow results in various serious complications including hypercalcemia, renal failure, anemia and bone lesions which are collectively referred to as CRAB features (Rajkumar et al., 2014; Rajkumar, 2020). Pathologically, MM is preceded by a premalignant phase termed monoclonal gammopathy of undetermined significance (MGUS), which is typically characterized by lower concentration of M proteins than that in MM (Ninkovic and Quach, 2020). MGUS is often asymptomatic, with or without an identified intervening stage, referred to as smoldering multiple myeloma (SMM). Most MGUS cases are clinically stable with a 1% annual risk of progression to MM (Kyle et al., 2018). Currently, there is a paucity of drugs that are able to recondition MM. Some new drugs, such as proteasome inhibitors bortezomib and carfilzomib as well as the immunomodulatory drugs (thalidomide, lenalidomide, and pomalidomide) have helped improve the treatment landscape for MM patients. However, the majority of patients ultimately develop recurrence. Therefore, development of novel therapeutic approaches for MM is a key imperative.

Natural products often constitute the basis for the identification of effective drugs. Many widely used drugs are of natural origin (Newman and Cragg, 2020). In other words, refinement, processing, and standardization of the active ingredients in natural products is still a common approach for the development of novel therapeutics. A recent study systematically reviewed anti-myeloma plant natural products including alkaloids, phenolics and terpenes. In addition, Traditional Chinese medicinal materials (TCMMs), as natural products, are also an attractive source of novel drugs for MM treatment. In recent years, a considerable body of scientific evidence has underlined the promising potential of bioactive ingredients derived from TCMMs for treatment of MM. To the best of our knowledge, there is no systematic summary of the anti-MM effects and clinical applications of these commonly utilized bioactive components extracted from TCMMs that are different from the natural products reviewed by Jöhrer et al.(Jöhrer and Ҫiҫek, 2021). The objective of this review was to systematically summarize the available evidence of the anti-MM effects of the bioactive components isolated from TCMMs and to identify future research priorities for the development of novel agents for MM.

## Active Components Derived From TCMMS for Multiple Myeloma

### Curcumin

**FIGURE 2:**
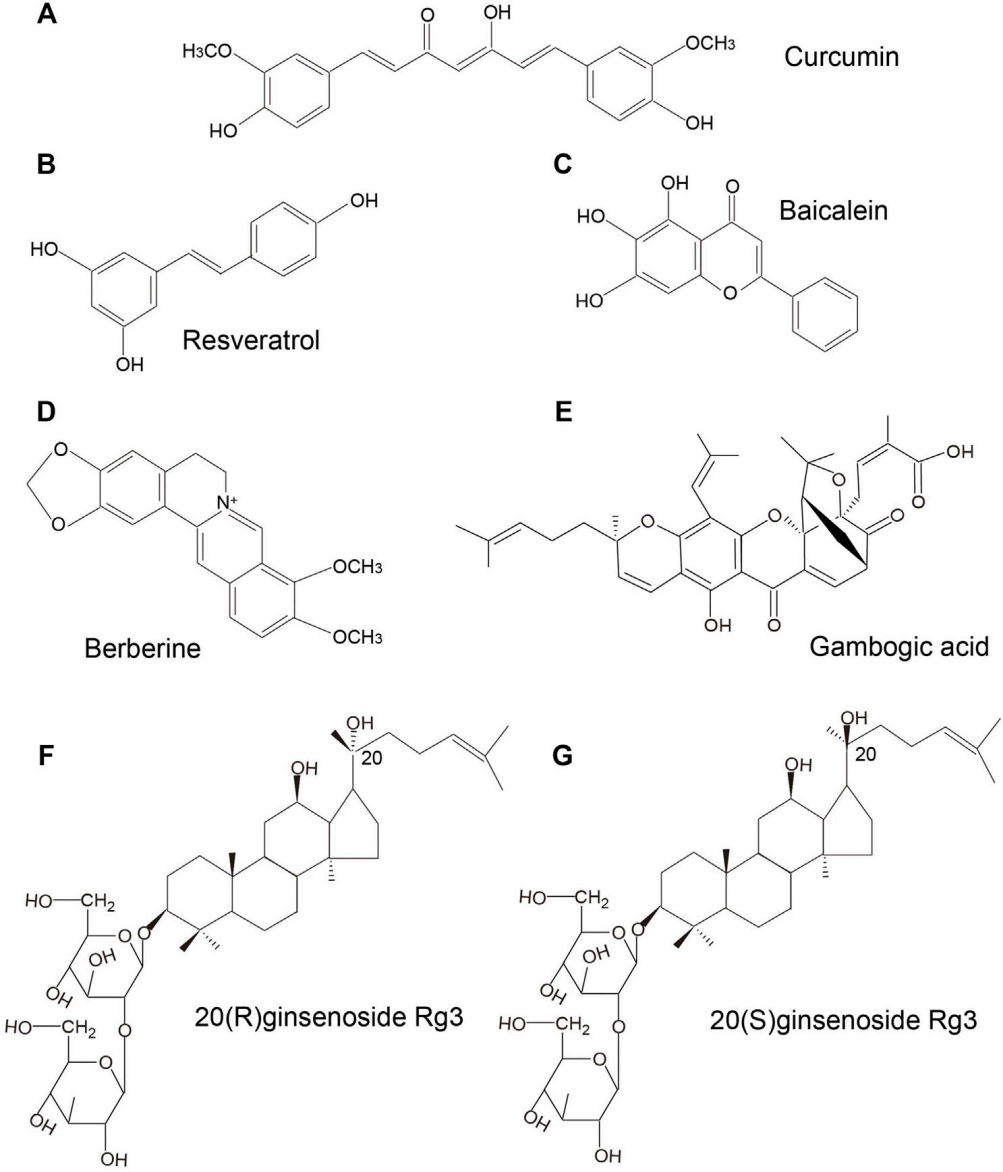
Chemical structures of active components extracted from TCMMs for treaing MM.

Curcumin, a polyphenol extracted from *Curcuma longa* (also known as turmeric) is widely used in medicine and as a dietary constituent worldwide. It has gained increasing attention for its greater bioactivity compared to the other bioactive compounds isolated from turmeric. Curcumin belongs to a chemical class of polyphenols with a chemical formula of C_21_H_20_O_6_ (Figure 2A). Curcumin exhibits a wide spectrum of pharmacological effects including anti-microbial, anti-inflammatory, anti-oxidant, anti-cancer, anti-viral, and neuroprotective effects. The mechanisms of action of curcumin against various cancers and other disorders have been intensively reviewed, such as breast cancer (Hu et al., 2019), colorectal cancer (Pricci et al., 2020), lung cancer (Wan Mohd Tajuddin et al., 2019), cerebral ischemia (Subedi and Gaire, 2021), neurodegenerative diseases (Monroy et al., 2013), and diabetes (Pivari et al., 2019). An increasing body of evidence from clinical and experimental studies has shown the great potential of curcumin as anti-MM therapy. In a study by Golombick et al., oral curcumin treatment (at a dose of 4 g daily) for 3 months led to a decrease in paraprotein load and bone resorption in patients with MGUS. However, only patients with a paraprotein of >20 g/L responded to curcumin (Golombick et al..,2009). Subsequently, results from a randomized, double-blind placebo-controlled trial suggested that curcumin treatment (4 and 8 g daily) may potentially slow down the disease progression in patients with smoldering MM (Golombick et al., 2012). In a recent clinical trial, patients with MM who received adjuvant curcumin treatment (at a dose of 3–4 g daily), as a replacement of dexamethasone due to intolerance to dexamethasone, showed decreased paraprotein load and plasmacytosis by 38 and 59%, respectively, when administered in combination with other anti-myeloma therapies. These findings indicated that curcumin may slow disease progression without inducing the adverse effects associated with steroid use (Ramakrishna et al., 2020). Besides, curcumin was shown to inhibit STAT3 phosphorylation in U266 MM cells in a dose- and time-dependent manner. Curcumin-induced inhibition of STAT3 phosphorylation not only suppressed the growth of myeloma cells, but also sensitized MM cells to dexamethasone (Bharti et al., 2003). A curcumin analog, FLLL332, was also found to specifically inhibit STAT3 phosphorylation and DNA binding activity, which resulted in inhibition of downstream target genes involved in cell proliferation including cyclin D1, Bcl-2, survivin, thus inducing apoptosis in MM cells (Lin et al., 2010). Another water-soluble curcumin analog, curcumin #12, was found to increase the sensitivity of MM cells to proteasome inhibitor bortezomib, thus inducing a considerable increase in caspase activity (Mujtaba et al., 2012). Curcumin was also shown to reverse the resistance to melphalan chemotherapy *in vitro* possibly via inactivation of the Fanconi anemia/BRCA pathway (Xiao et al., 2010). Allegra et al. (2018) reported that curcumin inhibited NF-κB pathway, upregulated expression levels of p53 and p21 that were implicated in the regulation of apoptotic pathways and cell cycle in U266 cells. A combination of curcumin and carfilzomib exerted stronger proapoptotic effects than curcumin or carfilzomib alone. Consistently, in several studies, curcumin was found to ameliorate chemoresistance and to sensitize MM cells to bortezomib via inhibiting Notch1 pathway (Ge et al., 2019) and NF-κB pathway (Sung et al., 2009), leading to the downregulation of cyclin D1, survivin, Bcl-xL, cIAP-1, XIAP, Bcl-2, TRAF1, and VEGF, which are associated with proliferation, apoptosis, and angiogenesis (Sung et al., 2009; Zhang et al., 2011). Anti-angiogenesis effects of curcumin were also confirmed by Wang et al.(Wang et al., 2006). They observed that curcumin inhibited angiogenesis via interrupting the interaction between MM cells and endothelial cells by decreasing TrkB expression in endothelial cells and suppressing BDNF production in MM cells. In addition, curcumin-induced epigenetic regulation on MM has also been investigated. Chen et al. (2019) found that curcumin induced apoptosis and suppressed the proliferation of MM cells via hypermethylating the promoter of mTOR in CpG sites, which in turn lead to an upregulation of mTOR signaling involved in the regulation of apoptosis and autophagy in MM.

**FIGURE 3:**
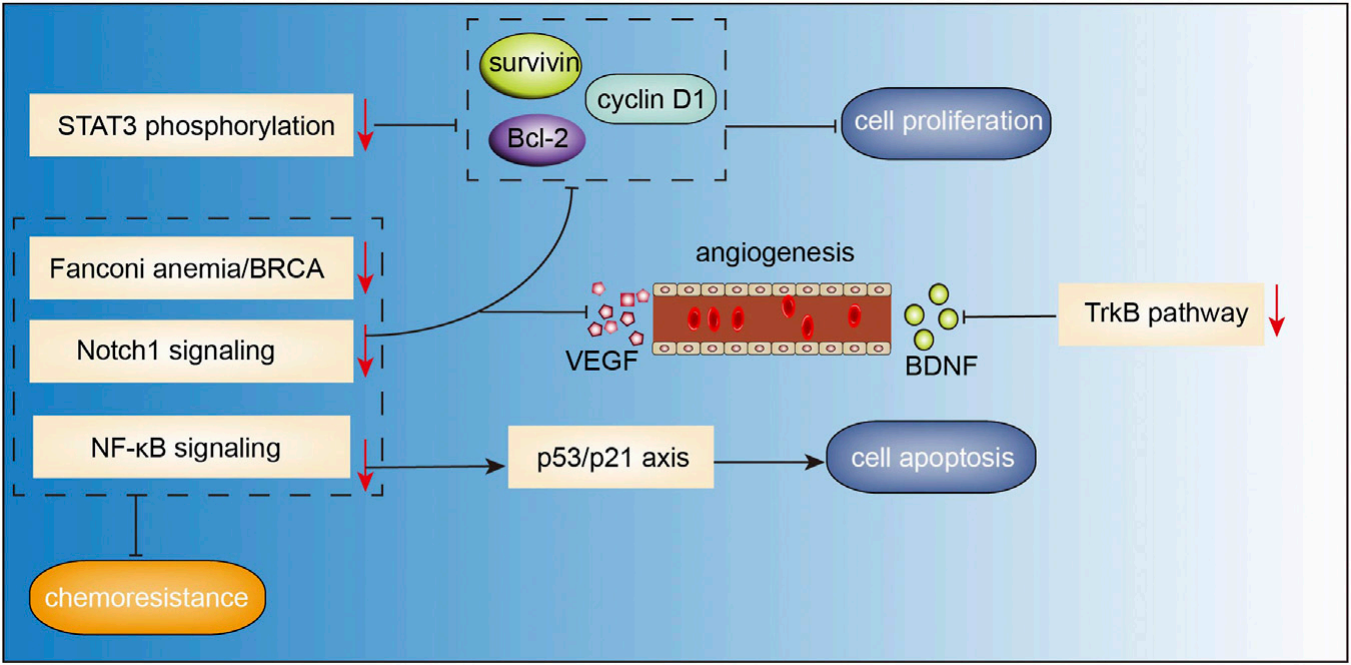
Action pathways involved in the anti-MM effects of curcumin. 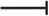
represents inhibitory effects, 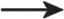
represents stimulative effects.

To summarize, the available evidence indicates multiple mechanisms of the anti-MM effects of curcumin, including induction of apoptosis, and inhibition of angiogenesis and proliferation (Figure 3).

### Resveratrol

**FIGURE 4:**
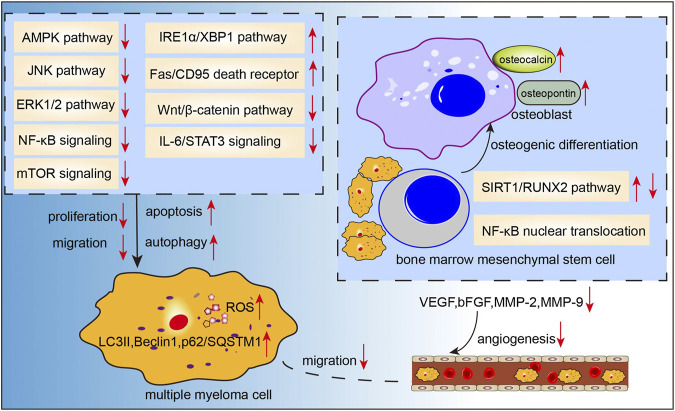
Action pathways involved in the anti-MM effects of resveratrol.

Resveratrol (3,4 ′,5-trihydroxy-trans-stilbene, Figure 2B) was first isolated from the roots of white hellebore in 1940s, and since then it has been extracted from a variety of other plant species. It was also abundantly found in the active ingredients isolated from several Chinese herbal medicines, such as *Polygonum cuspidatum* (Hu Zhang), *Semen cassia* (Jue Ming Zi), and *Veratrum nigrum* (Li Lu). Resveratrol has a broad spectrum of beneficial health effects including anti-aging, anti-oxidant, anti-inflammatory, neuroprotective, cardioprotective, anti-microbial, and immune-regulatory effects. Studies have shown that resveratrol may also exhibit significant anti-cancer activity against a wide range of solid tumors and hematological malignancies. Recently, the anti-MM effects of resveratrol have been gaining growing attention. In a study, resveratrol was found to suppress angiogenesis in RPMI 8226 cells by inhibiting the expressions of VEGF, bFGF, MMP-2, and MMP-9 (Hu et al., 2007), which was consistent with the results of a previous study (Sun et al., 2006b). In the study by Jin et al.(Jin et al., 2018), combination therapy with resveratrol and rapamycin exerted stronger effects in inhibiting proliferation and inducing apoptosis of MM cells, indicating that resveratrol may have an inhibitory effect on mTOR signaling. In another study, resveratrol was found to induce apoptosis of MM cells via mitochondrial apoptotic pathway and the recruitment of Fas/CD95 death receptor, and downstream signaling molecules into lipid rafts (Reis-Sobreiro et al., 2009). Wang et al.(Wang et al., 2011) demonstrated that resveratrol can induce endoplasmic reticulum stress response via activating IRE1α/XBP1 pathway, leading to a pro-apoptotic effects on MM cells. Additionally, it also suppressed pro-survival XBP1 signaling. Besides, inhibition of NEAT-1-mediated Wnt/β-catenin pathway (Geng et al., 2018), IL-6/STAT3 pathway (Bhardwaj et al., 2007), NF-κB signaling (Sun et al., 2006a; Bhardwaj et al., 2007), cyclin D1 and Bcl-xL (Sun et al., 2006a; Bhardwaj et al., 2007), ERK1/2 and JNK pathways (Xie et al., 2016) may also be involved in the resveratrol-induced suppression of cell proliferation and invasion, cell-cycle arrest, and apoptosis induction in MM. Other than the pro-apoptosis effects, resveratrol also showed pro-autophagic activities. Ma et al. (2021) found that resveratrol caused dose-dependent upregulation of the levels of LC3 and Beclin1 in several MM cell lines; further investigation demonstrated that resveratrol-induced autophagic flux was mediated by increasing the phosphorylation of AMPK at Thr172 site and decreasing the phosphorylation of mTOR (Ser2448), p70S6K(Thr389), and 4EBP1(Thr37/46). In addition, resveratrol not only sensitized the proteasome inhibitor carfilzomib-induced apoptosis via increasing the production of reactive oxygen species (ROS) in multiple kinds of MM cell lines, but also induced autophagy when administered in combination with low-dose carfilzomib, as evidenced by increased levels of LC3-II and p62/SQSTM1 (Li et al., 2018). Increased osteoclast formation and bone resorption and absence of bone formation are the serious consequences of MM (Terpos et al., 2018). In a study, resveratrol treatment was shown to promote osteogenic differentiation of bone marrow mesenchymal stem cells from patients with multiple myeloma via activating the SIRT1/RUNX2 pathway (Pan et al., 2021). Moreover, resveratrol inhibited osteoclastogenesis via receptor activator of nuclear factor-κB (NF-κB) ligand (RANKL)-induced osteoclast differentiation, which was associated with downregulation of RANK expression and decrease in NFATc1 stimulation and NF-κB nuclear translocation; these findings suggested that resveratrol may be an efficient osteoclast inhibitor in MM. Besides, resveratrol also induced the expressions of osteoblast markers osteocalcin and osteopontin in human bone marrow mesenchymal stem cells and sensitized their response to 1,25(OH)_2_ vitamin D_3_ (Boissy et al., 2005). Collectively, these studies demonstrate that the anti-tumor effects of resveratrol in MM cell lines include inhibition of cell proliferation, upregulation of apoptosis and autophagy, induction of oxidative stress, cell cycle arrest, and suppression of osteoclastogenesis (Figure 4). However, results from a phase-two study of resveratrol plus bortezomib for patients with relapsed and or refractory MM indicated minimal efficacy of resveratrol treatment; in addition, resveratrol caused severe adverse events such as nausea, vomiting, and even unexpected renal failure (Popat et al., 2013). Till date, there have been few clinical trials of resveratrol therapy in MM patients. Whether resveratrol is a potential therapeutic intervention for MM patients or not warrants further investigation.

### Baicalein

**FIGURE 5:**
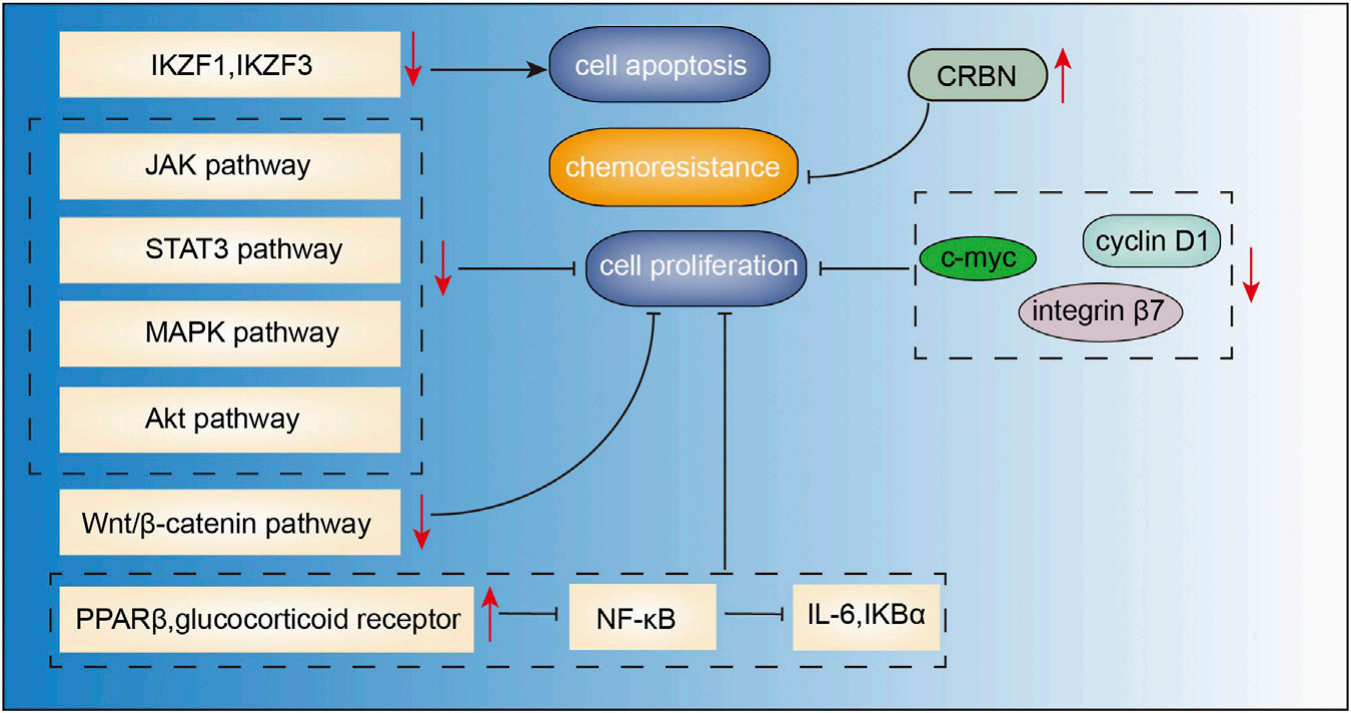
Action pathways involved in the anti-MM effects of baicalein. 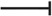
represents inhibitory effects, 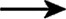
represents stimulative effects.

Baicalein (4H-1-benzopyran-4-one,5,6,7-trihy-droxy-2- phenyl-C_15_H_10_O_5,_ Figure 2C) is a major bioactive flavone derived from the roots of *Scutellaria baicalensis georgi* (Hunag Qin). Studies have demonstrated the anticancer effects of baicalein in the context of various cancers, including breast cancer (Yan et al., 2018), lung cancer (Zhang et al., 2020b), colorectal cancer (Phan et al., 2020), and hepatocellular carcinoma (Bie et al., 2017). Baicalein was shown to induce DNA damage without causing serious chromosomal instabilities or mutagenesis that may lead to severe side effects during chemotherapy (Fox et al., 2012). Recent studies have demonstrated the anti-MM effects of baicalein mediated via inhibition of proliferation and migration, and induction of apoptosis. In the study by Liu et al. (2018), baicalein suppressed the growth and promoted apoptosis of myeloma U266 cells via downregulating IKZF1 and IKZF3 (two essential lymphoid transcription factors in MM). Similarly, baicalein not only inhibited myeloma cell proliferation and induced apoptosis through downregulating IL-6 (Ma et al., 2005), but also abrogated IL-6-mediated signaling cascades including JAK, STAT3, MAPK, and Akt pathways associated with the proliferation and survival of MM cells (Liu et al., 2010). In addition, baicalein inhibited the proliferation and migration of RPMI 8226 and U266 MM cell lines via inhibition of Wnt/β-catenin pathway, c-myc, cyclin D1 and integrin β7, which are involved in cell proliferation (Xu et al., 2012). Otsuyama et al. (2007) found that combination of baicalein and dexamethasone can suppress the growth of U266 MM cells via activating PPARβ and glucocorticoid receptors, thus inhibiting the transcriptional activity of NF-κB followed by decreased levels of IL-6 and IKBα. Besides, baicalein can increase the sensitivity of MM cells to immunomodulatory drugs (IMiDs) by upregulating the CRBN which is also the target protein of IMiDs (Zhang et al., 2013). Side population cells show proliferation and differentiation potential similar to cancer stem cells. Gu et al. (2014) reported that baicalein decreased the proportion of side population cells via inhibition of ATP-binding cassette, subfamily G, isoform two protein (ABCG2) which is responsible for drug resistance in human MM cell line RPMI 8226. These findings suggest that baicalein may potentially target cancer stem cells of MM, indicating its potential use for the treatment of MM (Figure 5).

### Berberine

**FIGURE 6:**
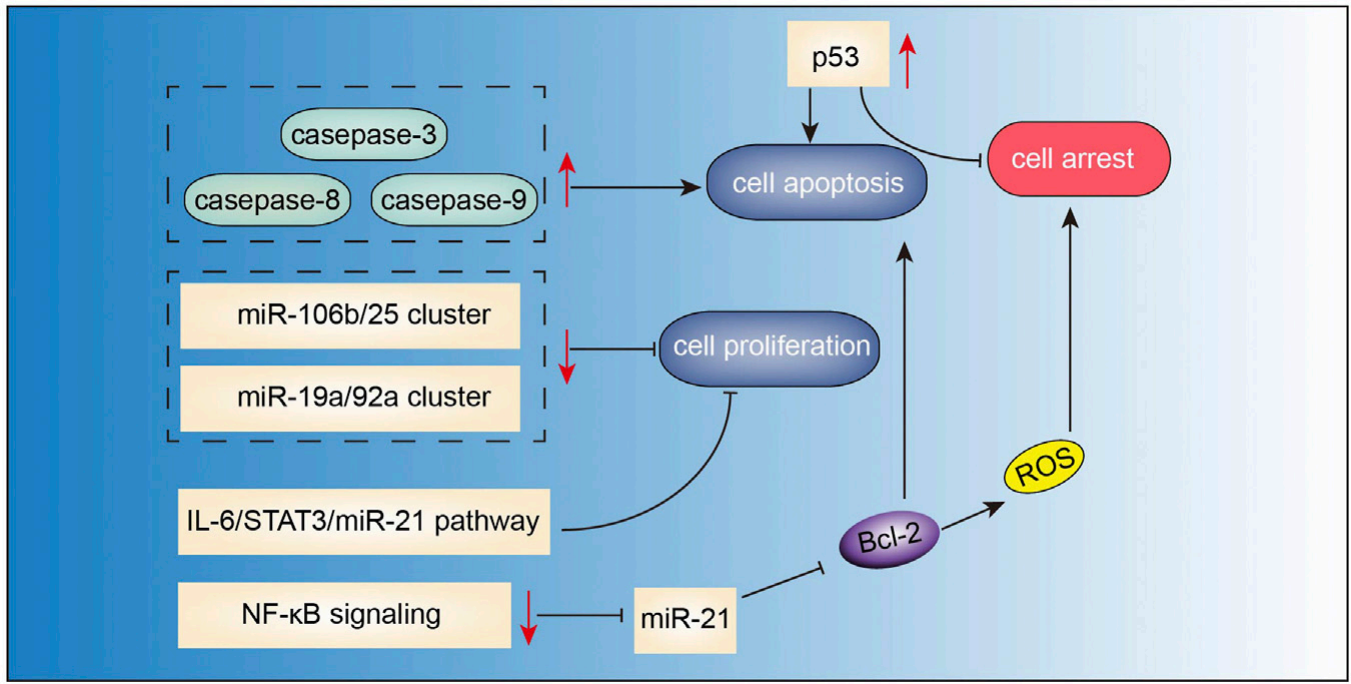
Action pathways involved in the anti-MM effects of berberine. 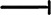
represents inhibitory effects, 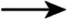
represents stimulative effects.

Berberine (Figure 2D) is the main active component of *Optidis rhizome* (Huang Lian). Several studies have demonstrated the anti-inflammatory, anti-oxidative, anti-microbial, anti-tumor, and neuroprotective effects of berberine. Recent experimental studies have also suggested the potential benefits of berberine in the treatment of MM (Figure 6). In the study by Tian et al., berberine administered in combination with bortezomib significantly increased the expressions of casepase-3, casepase-8, and casepase -9 in U266 cell line, and showed stronger pro-apoptotic effect than berberine or bortezomib alone (Tian et al., 2016). In a study by Qing et al.(Qing et al., 2014), berberine decreased the p53 DNA CpG methylation level via suppressing DNA methyltransferases DNMT1 and DNMT3B, and affected mRNA levels of several primary apoptosis-related proteins, thus inducing apoptosis and cell cycle arrest in U266 MM cells. Berberine can also suppress MM cells via possibly downregulating miRNA clusters including miR-99a∼125b, miR-17–92 and miR-106–25, which possibly affected MAPK, ErbB, and TP53 signaling pathways (Feng et al., 2015). Further studies validated that miR-106b/25 cluster encoding miR-106b, miR-93, and miR-25 (Gu et al., 2017), and miR-19a/92a cluster (Yin et al., 2018) were involved in berberine-induced inhibition of MM cells. Luo et al. (2014) found that berberine treatment suppressed MM cell growth via inhibiting IL6/STAT3/miR-21 pathway, which led to increased expression of PDCD4 and inhibition of p53 signaling. Similarly, berberine inhibited NF-κB translocation via Set9-mediated lysine methylation, resulting in a decrease in miR-21 level followed by decrease in Bcl-2 level, thereby triggering ROS generation and apoptosis in U266 MM cells (Hu et al., 2013). Lastly, Gu et al. (2020) also demonstrated that berberine treatment inhibited the growth of several MM cell lines via targeting UHRF1 (ubiquitin-like with PHD and RING Finger domains 1). Further molecular docking and surface plasmon resonance analysis confirmed UHRF1 as a berberine-binding protein and revealed that berberine binded UHRF1 in the tandem tudor domain and plant homeodomain.

### Chansu

Chansu, also known as toad venom, is an expensive TCMM extracted from the skin or parotid venom glands of *Bufo gargarizans* Cantor (Chen et al., 2018). Bufadienolides (including bufalin, cinobufagin, and resibufogenin) are considered as the main bioactive ingredients of Chansu. Among these, bufalin and cinobufagin are the main agents contributing to the antitumor effects (Zhang et al., 2020a). Studies have shown that Chansu can induce apoptosis and inhibit proliferation of MM cells. In the study by Wu et al. (2018), both bufalin and BF211 (a derivative of bufalin) were shown to suppress the progression of MM by inhibiting the IL-6/JAK2/STAT3 pathway *in vivo* and *in vitro*. Inhibition of IL-6/JAK2/STAT3 signaling pathway by BF211 led to increased levels of caspase-3, caspase-8, suppression of the expressions of Bcl-2 and Mcl-1, and showed stronger pro-apoptosis effects than bufalin. In addition, the cytocidal effect of bufalin in different MM cell lines was also mediated via inhibition of Akt/mTOR (Xiang et al., 2017). Results from Huang et al. (2013) revealed that the active site of bufalin interacted with the catalytic domain of ploy (ADP-ribose) polymerase1 (PARP1), triggering decreased activity of PARP1 and cell apoptosis as well as G_2_-M phase cell cycle arrest in various MM cell lines. While PARP1 overexpression reversed bufalin-induced cell apoptosis. Besides, cinobufagin was shown to exhibit pro-apoptotic effects in U266 cells through activating ERK, JNK and p38MAPK pathways (Baek et al., 2015). It should be noted that Chansu has a complex chemical composition, and its bioactive ingredients are highly toxic and may cause serious adverse events such as cardiac dysarythmia, tissue ischemia, and hypoxia (Li et al., 2020). Whether Chansu is suitable for the treatment of MM warrants further investigations.

### Gambogic Acid

**FIGURE 7:**
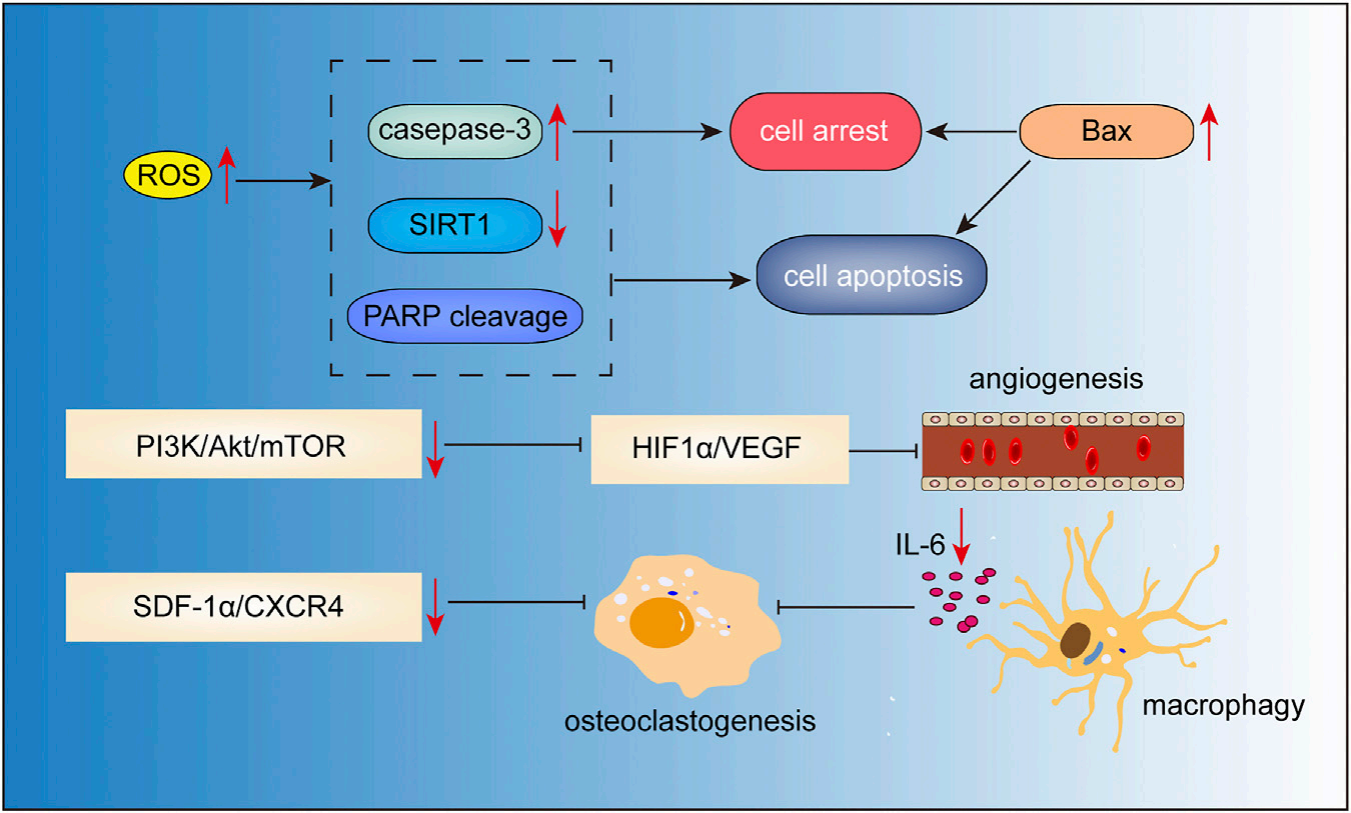
Action pathways involved in the anti-MM effects of gambogic acid. 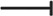
represents inhibitory effects, 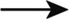
represents stimulative effects.

Gambogic acid (Figure 2E) is the major active component of gamboge derived from the *Garcinia hanburryi* tree. In TCM, gamboge (Teng Huang) has been used for relieving trauma–induced swelling and pain for thousands of years. It has been reported to exhibit potent anticancer activity against certain solid tumors. Recent studies have indicated the therapeutic potential of gambogic acid for the treatment of MM (Figure 7). Yang et al. (2012) found that gambogic acid can induce apoptosis of RPMI-8226 cells via ROS accumulation followed by caspase-3 activation, PARP cleavage, and SIRT1 downregulation. Hypoxic conditions in the bone marrow microenvironment have been implicated in the progression of angiogenesis, and chemotherapeutic resistance in MM (Ikeda and Tagawa, 2021). This hypoxic condition may induce adaptive cellular responses mediated via hypoxia-inducible transcription factors (HIF) (Martin et al., 2011; Borsi et al., 2015). In the study by Wang et al. (2014), gambogic acid treatment suppressed MM progression and angiogenesis through inhibition of HIF1α/VEGF expression, which was associated with the inhibition of PI3K/Akt/mTOR pathway. Osteoporosis and lytic lesions are also common in patients with MM (Andrews et al., 2021). Stromal cell-derived factor 1α (SDF-1α)/CXC chemokine receptor 4 (CXCR4) signaling has been shown to be associated with osteoclastogenesis (Dong et al., 2016). Gambogic acid was shown to inhibit SDF-1α-induced chemotaxis of MM cells and downstream signaling of CXCR4. Further study demonstrated that the gambogic acid-induced inhibition of CXCR4 was caused by inhibiting the binding of NF-κB to the CXCR4 prompter, which in turn caused inhibition of IL-6 expression in MM cells. Moreover, gambogic acid also inhibited expression of IL-6 in macrophages, thus inhibiting osteoclastogenesis (Pandey et al., 2014). Besides, gambogic acid and bortezomib at non-toxic concentration loaded with nanoparticles exhibited significant inhibitory effects by inducing G_2_/M phase cell cycle arrest and apoptosis via increasing expression of proapoptotic Bax, Caspase-3, and inhibiting anti-apoptotic PI3K/Akt pathway and Bcl-2 (Zhang et al., 2015).

### Ginsenoside

Ginseng (Ren Shen) is a famous traditional Chinese medicine herb used in China for more than two thousand years. The medicinal value of Ginseng has been recognized globally. Rg3, one of the pharmacologically active ginsenoside saponins isolated from ginseng has shown anti-aging, anti-inflammatory, anti-tumor, cardioprotective and neuroprotective properties. Ginsenoside Rg3 has been studied for the different efficacy of two chemical forms, namely 20(R)-ginsenoside Rg3 (Figure 2F) and 20(S)-ginsenoside Rg3 (Figure 2G). Recent studies have shown the therapeutic potential of ginsenoside Rg3 in MM. Song et al.(Song et al., 2014) reported that 20(S)-ginsenoside Rg3 suppressed the proliferation of U266 cells via partly inducing G_1_ phase cell cycle arrest and apoptosis, as evidenced by increased levels of caspase-3, caspase-8, and caspase-9. In addition, the VEGF secretion by U266 cells was also downregulated. Li et al.(Li et al., 2016) demonstrated that the inhibitory effects of Rg3 on the proliferation of MM cells were associated with inhibition of the IGF-1/Akt/mTOR signaling pathway. Besides, ginseng, compound K, a metabolite of the ginsenoside was also shown to induce apoptosis in U266 cells via inhibiting the JAK1/STAT3 pathway (Park et al., 2011).

### Other Bioactive Components

Other than the active ingredients discussed above, there are other bioactive components derived from TCMMs which have not been studied intensively, but could still provide new therapeutic options for MM patients. Icariin, a highly-potent active ingredient extracted from *Epimedium* (Ying Yang Huo), is a promising lead compound that has shown high efficiency in the treatment of various cancers. Jung et al.(Jung et al., 2018) recently reported that icariin significantly potentiated the apoptotic effects of bortezomib via inhibiting JAK/STAT3 signaling pathway, and downregulating the downstream targets including Bcl-2, Bcl-xl, survivin, IAP-1/2, COX-2, VEGF, and MMP-9. Additionally, daidzin, extracted from *Pueraria lobate* (Ge Gen) showed inhibitory effects on STAT3 signaling cascade in MM cells; this agent is frequently used to treat a broad spectrum of disorders including pain, diabetes, neurodegenerative diseases, gastrointestinal diseases, cerebrovascular disorders, and cardiac dysfunction (Wang et al., 2020). Formononetin, another naturally-occurring isoflavone derived from *Pueraria lobata* and *Astragalus membranaceus* (Huang Qi), can also suppress the DNA binding capacity and nuclear translocation of STAT3 and STAT5, thus triggering cell cycle arrest, inhibition of angiogenesis and proliferation, and induction of apoptosis in U266 and RPMI 8226 cells (Kim et al., 2018). *Hedyotis diflfus* (Bai Hua She She Cao) is a common ingredient in Chinese herbal medicine formulas used for cancer treatment. Lin et al.(Lin et al., 2013) found that polysaccharides extracts from *Hedyotis difus* significantly suppressed the proliferation and induced apoptosis in RPMI 8226 cells, at least, partly via downregulating Akt and NF-κB signaling cascades. Scutellarein is a flavonoid derived from *Scutellaria barbata* (Ban Zhi Lian), a famous and expensive anti-cancer traditional Chinese herbal medicine. It was shown to induce mitochondrial-mediated intrinsic apoptosis in a variety of MM cell lines, and to greatly reduce MM xenograft tumor burden in nude mice (Shi et al., 2019).

## Summary, Conclusion, and Future Perspective

Currently, bioactive components derived from TCMMs have been gradually drawing attention, and their cytotoxic effects on MM have been intensively validated. Collectively, the available evidence supports that curcumin, resveratrol, baicalein, berberine, bufadienolides, gambogic acid, ginsenoside, and other less investigated ingredients exhibit significant anti-MM effects via mainly suppressing angiogenesis and proliferation, by inducing cell apoptosis, cell cycle arrest and autophagy, as well as by inhibiting osteoclastogenesis. These findings indicate a promising role of bioactive components extracted from TCMMs as novel therapeutic agents for MM. However, several issues still need to be considered. First, studies that have investigated the cytotoxic effects of these ingredients on MM cell lines have largely been limited to assessing the effects on cell proliferation, apoptosis, or autophagy, while there is a paucity of investigations on the immune system or multiple myeloma bone marrow microenvironment. In addition, epigenetic modifications induced by these ingredients should also be studied since epigenetics modulation are risk factors including environmental and occupational exposures, as well as aging (Dimopoulos et al., 2014). Moreover, theses bioactive components may have a cytotoxic effect on normal cells, while most studies have been performed on MM cells. Whether and how these ingredients discriminate between normal and MM cells remains unknown. Secondly, the targeting proteins, metabolites, and signaling pathways involved in the regulation of angiogenesis, proliferation, apoptosis should be studied by utilizing multi-omics technologies, including genomics, transcriptomics, proteomics, and metabolomics. This approach may provide more in-depth characterization of the mechanisms of actions of these bioactive components. Besides, network pharmacology and molecular docking analysis on molecular targets should also be considered to shed light on the underlying mechanisms of TCMMs. Additionally, the anti-MM impact of promising ingredients, such as curcumin and resveratrol, in the clinical context is still quite vague. Few clinical studies have evaluated treatment with these ingredients alone or in combination with other drugs in MM patients. Thus, further well-designed clinical trials with follow-up and *in-vivo* examinations should be performed. Third, many of these ingredients exhibit poor bioavailability and are rapidly metabolized, which may undermine their pharmacological activity. Therefore, discovery and utilization of drug delivery systems, such as nanoparticles and liposomes, as well as the novel synthetic analogs can enhance drug stability, prolong drug action, and enable a sustained and slow-release rate while reducing the side effects.
